# Zoos through the Lens of the IUCN Red List: A Global Metapopulation Approach to Support Conservation Breeding Programs

**DOI:** 10.1371/journal.pone.0080311

**Published:** 2013-12-11

**Authors:** Dalia A. Conde, Fernando Colchero, Markus Gusset, Paul Pearce-Kelly, Onnie Byers, Nate Flesness, Robert K. Browne, Owen R. Jones

**Affiliations:** 1 Max-Planck Odense Center on the Biodemography of Aging, University of Southern Denmark, Odense M, Denmark; 2 Department of Biology, University of Southern Denmark, Odense M, Denmark; 3 Centre for Research and Conservation, Royal Zoological Society of Antwerp, Antwerp, Belgium; 4 Department of Mathematics and Computer Science, University of Southern Denmark, Odense M, Denmark; 5 World Association of Zoos and Aquariums, Gland, Switzerland; 6 Zoological Society of London, London, United Kingdom; 7 IUCN SSC Conservation Breeding Specialist Group, Apple Valley, Minnesota, United States of America; 8 International Species Information System, Eagan, Minnesota, United States of America; Bangor University, United Kingdom

## Abstract

Given current extinction trends, the number of species requiring conservation breeding programs (CBPs) is likely to increase dramatically. To inform CBP policies for threatened terrestrial vertebrates, we evaluated the number and representation of threatened vertebrate species on the IUCN Red List held in the ISIS zoo network and estimated the complexity of their management as metapopulations. Our results show that 695 of the 3,955 (23%) terrestrial vertebrate species in ISIS zoos are threatened. Only two of the 59 taxonomic orders show a higher proportion of threatened species in ISIS zoos than would be expected if species were selected at random. In addition, for most taxa, the management of a zoo metapopulation of more than 250 individuals will require the coordination of a cluster of 11 to 24 ISIS zoos within a radius of 2,000 km. Thus, in the zoo network, the representation of species that may require CBPs is currently low and the spatial distribution of these zoo populations makes management difficult. Although the zoo community may have the will and the logistical potential to contribute to conservation actions, including CBPs, to do so will require greater collaboration between zoos and other institutions, alongside the development of international agreements that facilitate cross-border movement of zoo animals. To maximize the effectiveness of integrated conservation actions that include CBPs, it is fundamental that the non-zoo conservation community acknowledges and integrates the expertise and facilities of zoos where it can be helpful.

## Introduction

The conservation status of known biodiversity has undergone a worrying decline in the last few decades [1], [2]. If the present trends continue, the conservation community will be challenged with a large number of species for which there is no viable conservation outcome. As a result, conservation breeding programs (CBPs) may offer the only feasible option to avoid the extinction of particular species until appropriate habitat can be found or restored [3]–[5]. The need for well-managed CBPs, including those supported by the zoo community, has never been greater, but the space and logistical demands for managing CBPs could exceed the current capacity of the zoo community [1], [5]. Although we know that 15% of threatened vertebrate species are represented in zoos [3], we still do not have an overview of the number and distribution of these species within the global zoo network. Therefore, to establish effective responses to species' CBPs, it is important to determine the current representation of threatened species within zoos and have a general understanding of the complexity of managing them within the global zoo network.

The development of CBPs is challenging and has received criticism due to problems including hybridization risk, high costs and the diversion of resources away from habitat protection [6]–[9]. Because of these factors, CBPs have usually been considered only as a last resort and, as a result, are normally only implemented when populations dwindle to fewer than 20 individuals (e.g. whooping crane, *Grus americana* [10]). Still, CBPs have played a major role in 13 of the 68 species that have shown a status improvement in IUCN Red List reassessments [11], [12]. Moreover, for mammals, captive breeding together with reintroduction programs and hunting restriction have been the most effective conservation actions, since ensuring protected areas alone has not been enough [13]. As a result, in most cases effective conservation plans require the integration of a range of management options where CBPs could be necessary. For example, Martin et al. [14] compared the failure of conservation intervention for the recently extinct Christmas Island pipistrelle (*Pipistrellus murrayi*) with the potentially successful intervention in the case of the orange-bellied parrot (*Neophema chrysogaster*), where conservation actions, including CBPs, were implemented in a timely manner and coordinated among several institutions.

As threats to biodiversity increase, the management of at-risk species requires a spectrum of interventions that can range from habitat protection to the establishment of CBPs with the eventual aim of reintroduction into the wild [3], [4]. Therefore, differentiating between *in situ* and *ex situ* management is becoming irrelevant [4], [15]. Instead, a continuum of management practices exists, which ranges from truly wild and self-sustaining populations to managed populations dependent on a certain level of human care for persistence. For example, the IUCN SSC Conservation Breeding Specialist Group has recently launched an ambitious conservation framework called the One Plan approach, which promotes participation between different institutions and stakeholders with the aim to consider all populations of a particular species, both inside and outside their natural range, under a unified management plan [16]. The zoo community is in an ideal position to support and further contribute to develop such conservation programs because they are involved in both captive breeding and field conservation programs. For example, zoos have extensive knowledge of the husbandry, behavior and veterinary procedures required to develop CBPs [17], and members of the World Association of Zoos and Aquariums (WAZA) are, collectively, the third largest financial supporter of species conservation in their natural habitats (providing US$350 million/year), while also being actively involved in many of those projects [18].

To understand the space devoted to threatened species and the potential complexity of managing CBPs as zoo-held metapopulations, we evaluated the representation of the IUCN Red List threatened species held within the zoo network. We used data published from Conde et al. [3], processed from the International Species Information System (ISIS) organization. ISIS is a network of more than 800 zoos and aquariums that shares information about ∼2.5 million individuals among the member institutions. This information system makes it possible to analyze the amount of space devoted to threatened species and its spatial distribution. Therefore, ISIS is a key institution to assess the potential of zoos to develop CBPs. To understand if the number of threatened species in zoos is the result of a sound prioritization or if it has been more an opportunistic process, we analyzed whether the representation of threatened species per taxonomic order is significantly higher to what would be expected if species were randomly collected (i.e. without collection planning). In addition, to appraise the approximate complexity of managing zoo-held species as metapopulations, we estimated the distance between zoos clusters at which the probability of reaching an average metapopulation size of 50, 100 and 250 individuals is maximized. With these analyses we can better infer how complex it is to reach increasing population sizes between ISIS zoos, assuming that CBPs could be more successful when population sizes are larger and clustered in zoos at closer proximity. Since the clustering of zoos could facilitate special treaties to move individuals across borders. Based on our results, we discuss the potential of zoos to contribute towards CBPs for terrestrial vertebrates and make some policy recommendations.

## Methods

### Representation of threatened species by order in the ISIS network

To determine whether the number of threatened species in ISIS zoos is different from what would result from a random sample, we carried out the following analyses. For every taxonomic order *i* of terrestrial vertebrates there is a given number *n_i_* of species worldwide, out of which a fraction *m_i_* is known to be threatened. Similarly, we know the number of species for every order *z_i_* that are represented in ISIS zoos and the subset *w_i_* of those that are threatened. We developed a Monte Carlo algorithm to understand which orders have a number of threatened species in ISIS zoos significantly different from a random sample of species in the wild. This algorithm was based on an iterative procedure that, at every step *s*, randomly sampled, without replacement, *z_i_* species within every order from the worldwide list. From these randomly sampled species, the algorithm counted the number of species 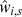 classified as threatened. The algorithm then calculated the indicator *y_i,j,s_* that assigned 1 if a number *j* of threatened species were sampled and 0 otherwise, such that *j* = 0, …, min(*z_i_*, *m_i_*). The probability of randomly finding *j* threatened species is calculated as
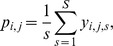
where *S* is the total number of iterations. Since the algorithm ran for several thousand iterations, we were able to construct a distribution (i.e. empirical probability mass function, PMF) of the number of threatened species per order that could have been found in ISIS zoos if they had sampled species at random from the wild. The probability in the PMF that corresponds to the real number of threatened species in zoos (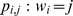) was used as an analogue to the *P*-value. Those orders that had a number of threatened species that matched the lower bounds of the PMF (i.e. *P*-value of 0.05) were classified as being significantly under-represented. Similarly, those that matched upper bounds of the PMF (i.e. above the 0.95 quantile) were catalogued as being over-represented. The remaining species could not be distinguished from a random sample.

### Managing metapopulations of threatened species in the ISIS network

To understand the complexity of maintaining CBPs of threatened species across the ISIS zoo network, we developed a second algorithm to find the optimal radial distance from any given zoo at which the probability of finding a metapopulation size of at least 50, 100 or 250 recruited living individuals was highest relative to that radial distance. The algorithm was based on a Monte Carlo procedure that, at each step *s*, a zoo *j* was selected at random, then found all zoos *z_j_*_,*r*_ located at a distance *r* from zoo *j*, as well as the number of zoos 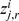 within that radial distance that held species *i*. We labeled the zoos included within that radius as a *cluster*, *K_j,r_*. The algorithm then counted how many individuals of a given threatened species *i* were included in the cluster, such that
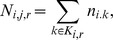
where *n_i,k_* is the population size for species *i* in zoo *k*. At each cluster, we assigned the indicator
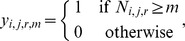
where *m* is a pre-established *metapopulation* threshold (i.e. 50, 100 or 250 individuals). We chose 50, 100 and 250 individual metapopulation thresholds because a population size of 50 has been historically considered as a minimum viable population [19], and 250 individuals is the threshold defined by the IUCN Red List as a “very small wild population” in the Critically Endangered category [20]. Moreover, very few threatened species in ISIS zoos have populations for which a higher threshold is possible. For instance, only 44 species (6.3%) have more than 1,000 individuals (see below). We considered 100 individuals as an arbitrary intermediate between these two values.

This procedure was repeated for 2,000 iterations and then the algorithm was repeated, increasing the radial distance by 100 km until a maximum radius of 10,000 km was reached. From the 2,000 iterations for each radius, the algorithm calculated the probability of finding at least *m* individuals for species *i* and radius *r* as
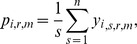
where *S* is the total number of steps (2,000) in the Monte Carlo procedure. Here, we replaced the subscript *j* with *s* to indicate that zoos were chosen at random with replacement. Similarly, we calculated the average number of zoos for each species at each distance interval as
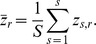



We repeated this procedure for radii ranging from 0 to 10,000 km, using 100 km increments. We excluded species for which the minimum population size was never reached, even when including all ISIS zoos.

For each class *C* and metapopulation threshold *m*, we calculated the average ratio between probability *p_i,r,m_* and distance *d_r_* for radius *r* as
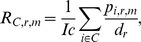
where *I_C_* is the total number of threatened species in class *C* that was included in the analysis. Finally, we found the *optimal cluster*, this is the optimal radius 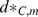 and the optimal number of zoos 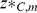, where the ratio *R_C,r,m_* was highest for each class and population threshold *m*. These optimal clusters imply that, relative to that radius, the probability of reaching a metapopulation of at least *m* individuals is highest. For simplicity, we present the results pooled by taxonomic class.

## Results

**Figure 1:**
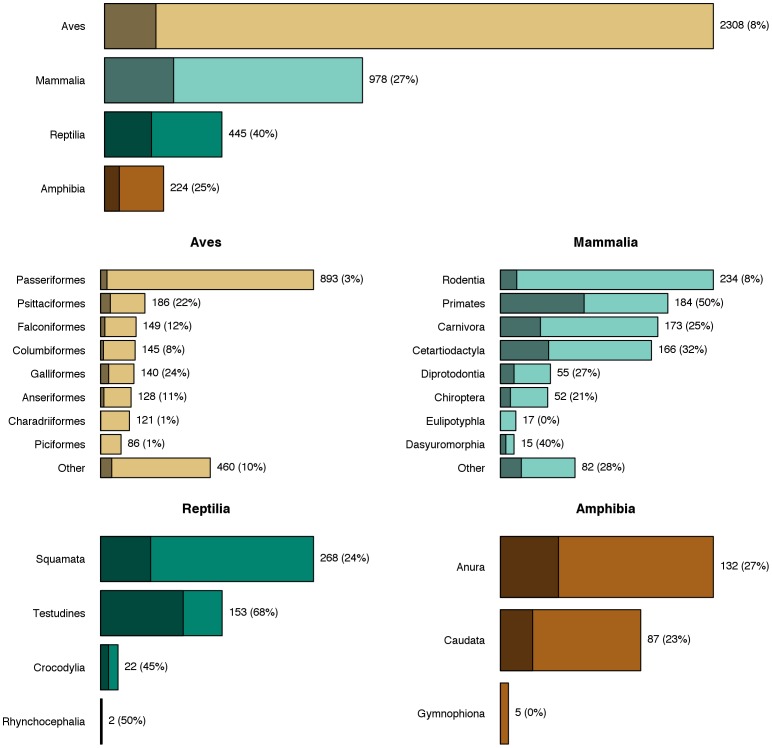
The representation of terrestrial vertebrate species held in ISIS zoos. The upper panel summarizes the representation of species for each taxonomic class of terrestrial vertebrates while the four lower panels summarize representation at the taxonomic order level within each class. The length of each bar is proportional to the number of species held, and each bar is color-coded by class. The darker shaded region of each bar represents the number of species that fall into the three IUCN Red List threatened categories (Vulnerable, Endangered and Critically Endangered). The number of species in each category is indicated to the right of each bar, and the percentage of these that are threatened is given in parentheses.

Collectively, in 2011, the 837 ISIS zoos held 3,955 species of non-domestic terrestrial vertebrates (Table 1). Of these, more than half (58%) were birds, one quarter (25%) were mammals, 11% were reptiles and 6% were amphibians (Figure 1). Twenty-three percent (691/3,955) of the species in ISIS zoos belong to a threatened category (Table 2). Within each class, the percentage of threatened species varies widely among each order, ranging between 8% for birds (195/2,308) and 27% for mammals (262/978). ISIS zoos held a total of 455,317 individuals of non-domestic terrestrial vertebrates, of which 22% (91,063/455,317) belong to a threatened species (Table S1).

**Table 1:** Number of species assessed in the IUCN Red List and number of species held in ISIS zoos for each taxonomic class of terrestrial vertebrates.

Class	Total species on Red List	Threatened species on Red List*	Total species in ISIS zoos	Threatened species in ISIS zoos
Mammalia	5,488	1,140	978	262
Aves	9,934	1,253	2,308	195
Reptilia†	3,663	802	445	178
Amphibia	6,370	1,917	224	56
Total	25,455	5,186	3,955	691

* The IUCN Red List refers to the categories of VU, EN and CR collectively as threatened species.

† The reptile assessment by IUCN is not yet complete, so these results only refer to the species assessed in the IUCN Red List version 3.2 (2009).

**Table 2:** Number of species of terrestrial vertebrates held in ISIS zoos broken down by IUCN Red List category.

Class	DD	LC	NT	VU*	EN*	CR*	EW	Total species in ISIS zoos	Threatened species in ISIS zoos
Mammalia	27	606	81	122	104	36	2	978	262 (27.0%)
Aves	1	1,956	152	115	62	18	4	2,308	195 (8.4%)
Reptilia†	10	207	50	87	43	48	0	445	178 (40.0%)
Amphibia	3	141	22	28	13	15	2	224	56 (25.0%)
Total	41	2,910	305	352	222	117	8	3,955	691 (23.3%)

* VU, EN and CR are collectively referred to as threatened species.

† The reptile assessment by IUCN is not yet complete, so these results only refer to the species assessed in the IUCN Red List version 3.2 (2009).

The seven IUCN Red List categories are DD = Data Deficient, LC = Least Concern, NT = Near Threatened, VU = Vulnerable, EN = Endangered, CR = Critically Endangered, EW = Extinct in the Wild. We further break down the accounting by taxonomic class. Note: In parenthesis are the percentages of threatened species with respect to the species present in ISIS zoos and not with respect to the total number of the world's threatened terrestrial vertebrate species (that information is available in Conde et al. [3]).

### Representation of threatened species by order in the ISIS network

**Figure 2:**
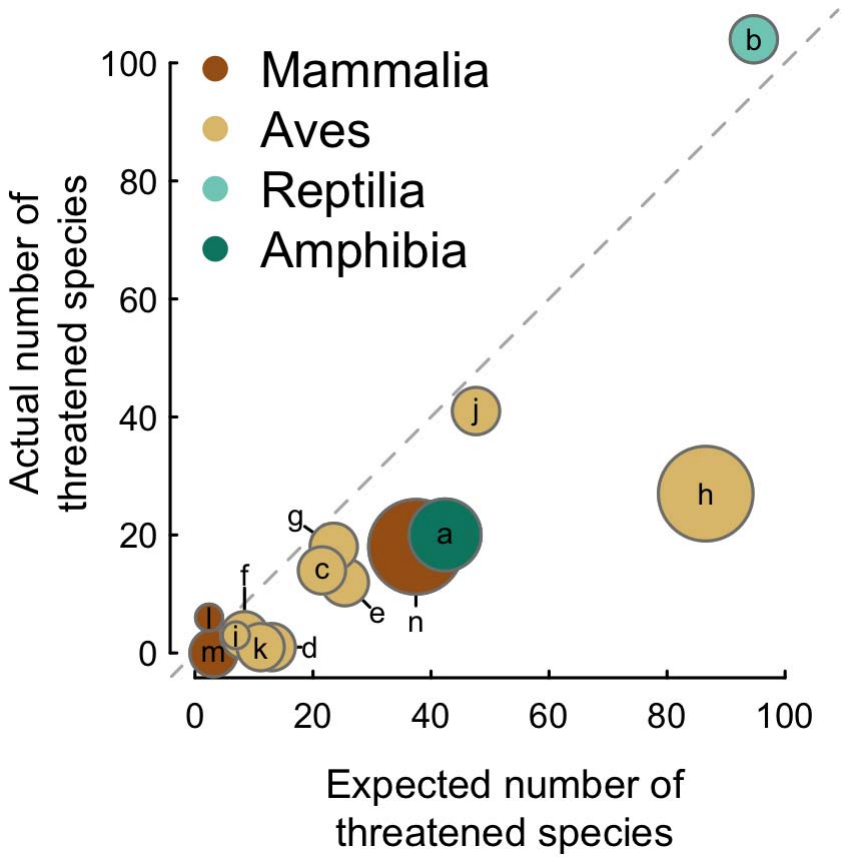
Expected versus actual number of threatened species per taxonomic order in ISIS zoos for each taxonomic class of terrestrial vertebrates. Each point represents an order that was significantly different from the expected value if zoo collections were taken at random. The size of the point corresponds to the number of threatened species for each order. Labels correspond to: a) Caudata; b) Testudines; c) Anseriformes; d) Charadriiformes; e) Columbiformes; f) Coraciiformes; g) Falconiformes; h) Passeriformes; i) Pelecaniformes; j) Psittaciformes; k) Strigiformes; l) Dasyuromorphia; m) Eulipotyphyla; n) Rodentia.

Our analysis of the number of threatened species in ISIS zoos, broken down by order, shows that most collections are not distinguishable from what would be expected if the species were selected at random (Figure 2, Tables S2, S3, S4, S5). Exceptions occur in mammals in the order Dasyuromorphia (Australian carnivorous marsupials) and in reptiles for Testudines (turtles): ISIS zoos hold 50% and 79%, respectively, of the order's threatened species. On the other hand, threatened species were under-represented in zoos for the mammalian orders Eulipotyphyla (insectivores) and Rodentia (rodents). For birds, threatened species were under-represented in nine of the 25 orders held in zoos, whereas for amphibians this was only the case for Caudata (salamanders).

### Managing metapopulations of threatened species in the ISIS network

For the threatened amphibian species in ISIS zoos, 27% reach a metapopulation size threshold of >50 individuals. Almost half of the threatened species of mammals, birds and reptiles in ISIS zoos reach the same threshold (44%, 47% and 43%, respectively), and 18% of the threatened mammals reach a threshold above 250 individuals (Table 3). However, many of those species are distributed among numerous zoos, thus if those species are not managed as a metapopulation, their conservation potential will be greatly reduced.

**Table 3:** Number of species in ISIS zoos with population sizes within specific thresholds for the different Red List categories (see Table 2 for Red List categories definition).

Class	Threshold (number of individuals)	DD	LC	NT	VU	EN	CR	EW
Amphibia	0–50	0	0	0	0	0	0	0
	50–100	3	101	14	22	10	9	0
	100–250	0	16	4	3	1	2	0
	250–500	0	10	1	1	0	0	0
	500–1,000	0	11	2	1	0	3	1
	1,000–2,000	0	3	1	1	1	0	0
	>2,000	0	0	0	0	1	0	0
Reptilia	0–50	0	0	0	0	0	0	0
	50–100	10	159	33	51	25	25	0
	100–250	0	20	4	9	12	8	0
	250–500	0	19	6	12	5	11	0
	500–1,000	0	6	3	10	1	3	0
	1,000–2,000	0	1	2	4	0	1	0
	>2,000	0	2	1	1	0	0	0
Aves	0–50	0	0	0	0	0	0	0
	50–100	0	1,455	96	69	31	10	3
	100–250	0	185	25	12	14	3	1
	250–500	1	179	18	23	12	3	0
	500–1,000	0	71	3	3	4	0	0
	1,000–2,000	0	40	6	4	1	1	0
	>2,000	0	17	3	2	0	1	0
Mammalia	0–50	0	0	0	0	0	0	0
	50–100	24	370	42	65	55	18	0
	100–250	0	66	10	18	13	3	0
	250–500	2	78	12	20	16	7	0
	500–1,000	0	46	8	10	11	3	0
	1,000–2,000	1	27	4	5	7	4	1
	>2,000	0	9	3	4	2	1	1

**Figure 3:**
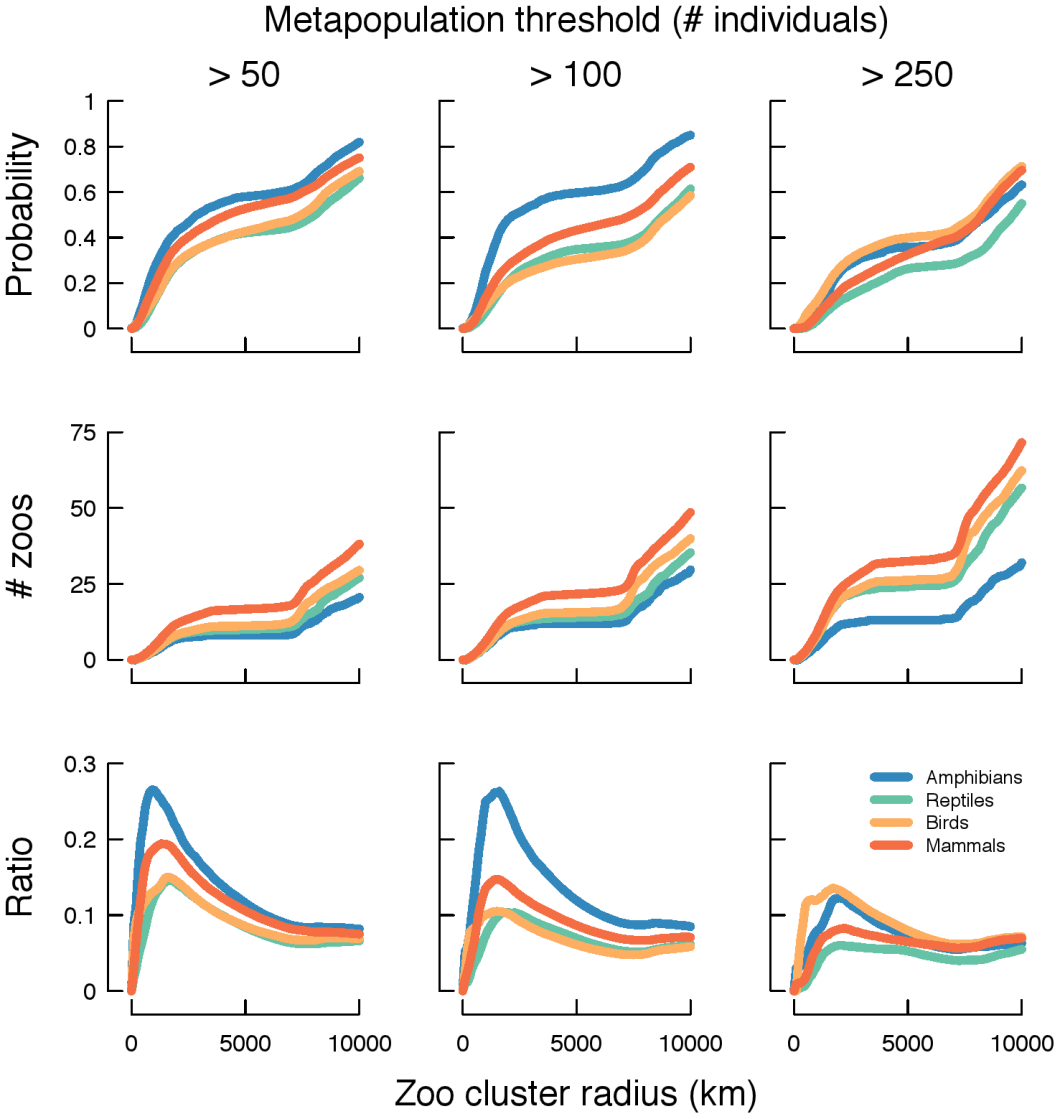
Average probability of finding a metapopulation size above 50, 100 or 250 individuals as a function of the length of the zoo cluster radial distance (upper row), average number of zoos where the species is found as a function of the radial distance (middle row) and ratio between the probability of finding the metapopulation and the radial distance (lower row) for each taxonomic class of terrestrial vertebrates. When the ratio is highest, we obtain the optimal radial distance between zoos and the optimal probability of finding a metapopulation size above the threshold. This is, at that ratio the probability of finding the metapopulation is highest with respect to the zoo cluster radial distance. For display purposes all ratios were multiplied by 1,000.

As expected, the optimal distance radii and the number of zoos required to maximize the probability of reaching a given population increase with the metapopulation size threshold (>50, >100 and >250 individuals) (Figure 3). However, the magnitude of the increase varies among classes; for birds and reptiles, the difference between optimal distances is just 200 km and 300 km, respectively, while for mammals and amphibians, the differences are up to 900 km (Table 4).

**Table 4:** Optimal radial distance between zoos in a metapopulation network for each taxonomic class of terrestrial vertebrates.

Class	Metapopulation threshold *m*	Number of species in cluster	Radial distance (km)	Probability of reaching *m*	Average number of zoos in cluster	Average number of zoos with species in cluster
Mammalia	>50	124	1,300	0.252	126.6	7.1
	>100	90	1,500	0.221	159.9	11.5
	>250	47	2,200	0.182	239.8	24.6
Aves	>50	85	1,600	0.240	175.2	6.6
	>100	56	1,500	0.158	160.1	8.5
	>250	18	1,700	0.231	187.2	16.9
Reptilia	>50	77	1,700	0.248	186.5	6.9
	>100	48	1,900	0.197	212.1	10.8
	>250	20	2,000	0.121	224.1	19.6
Amphibia	>50	15	900	0.239	69.8	2.5
	>100	9	1,600	0.421	174.6	8.1
	>250	8	2,000	0.245	224.1	11.3

Optimal radial distance between zoos, average probability of reaching the metapopulation size threshold within that radial distance, average number of zoos within radial distance and average number of zoos within radial distance that hold the threatened species. The optimal radial distance represents the distance needed to optimize the probability of reaching an average metapopulation size within the shortest possible distance between zoos (the metapopulation thresholds *m* are >50, >100 and >250 individuals). The column for “number of species in cluster” indicates the number of threatened species for which all ISIS zoos have at least *m* individuals.

Furthermore, at the optimal radial distances, the probability that a cluster of zoos has more than 50, 100 or 250 individuals of a given threatened species ranges from 0.16 to 0.42 (Table 4). However, only 3.5% to 10.2% of zoos within an optimized cluster have individuals of the species. Moreover, these percentages vary considerably among the four classes of terrestrial vertebrates.

## Discussion

### Representation of threatened species by order in the ISIS network

Birds and mammals make up the largest proportion of terrestrial vertebrates held in zoos. Although the majority of zoo collection plans were not originally focused on holding threatened species [21], 23% of their collections are currently devoted to them. However, for most of the taxonomic orders, our results show that representation of threatened species is not different from what would be expected if species were selected at random. Broken down by class, it is clear that threatened birds make up the lowest proportion in the zoo network. This may be explained by factors including: i) some species are difficult to breed in captivity (e.g. seabirds), ii) some species have specialized dietary requirements (e.g. insectivores), and iii) import/export restrictions, such as those added in the wake of the SARS (severe acute respiratory syndrome) epidemic, which make it challenging to manage birds across borders and to import new species.

Mammalian and reptilian zoo collections include the highest proportion of threatened species; however, only the orders Dasyromorphia and Testudines are significantly over-represented. The Dasyromorphia are a particular focus of CBP efforts in Australian zoos [22]. However, despite the interest in this group, six threatened Dasyromorphia species are not yet represented in zoos. The high representation of threatened Testudines is partly because many zoos serve as rescue centers for confiscated individuals. The Turtle Survival Alliance has played a key role in promoting the conservation of turtles and has been working on linking zoos and governmental institutions to ensure the rescue of animals from the illegal trade [23]. However, since many of those individuals come from the illegal trade, it is hard to include them as part of CBPs because, in many cases, their origins are uncertain [23].

For amphibians, the relatively low proportion of threatened Caudata in zoos may reflect their cryptic behavior and small size [24], which makes them difficult to display. In general, the small number of amphibian species may also be due to practical issues such as the difficulty in obtaining permits to transport individuals. Although amphibian collections in zoos and zoo-supported centers have significantly increased in the last 10 years [12], [25], zoos still only hold 3% of the world's threatened amphibians [3]. This emphasizes the need for zoos to increase their contribution towards amphibian CBPs either as part of their collections or by further contributing to the development of breeding centers in their local areas [26]. With this in mind, the Amphibian Ark emerged with a strong zoo component with the mission of ensuring the global survival of amphibians, focusing on those that cannot currently be safeguarded in nature and where zoos can play a key role [27].

### Managing metapopulations of threatened species in the ISIS network

Zoos hold ∼15% of the world's threatened terrestrial vertebrates [3]. However, in their collections, 23% of ISIS zoos species (691/3,955) belong to a threatened category. Nevertheless, most of their populations are small and are distributed across the zoo network. Therefore, for the zoo community, one of the main challenges of managing their threatened species in CBPs is the complexity of moving individuals across borders and the coordination of conservation efforts among zoos and other institutions at a global level. The enormity of this task is clear from our results. For example, the optimal radius for finding a metapopulation size of more than 250 individuals for a given threatened species is 1,700 km for reptiles and 2,200 km for mammals. Within these optimized clusters the number of zoos that hold a given threatened species is low compared with the number of zoos available within the clusters. For example, on average only 13% (17/227) of all the zoos within a cluster hold a given threatened reptile species. This implies that, under current conditions, to manage a metapopulation above 250 individuals requires an optimized cluster of ∼20 zoos that could be up to 4,000 km apart. Given this complexity, it is not surprising that most zoo populations of threatened species are not managed as metapopulations [28], nor that most are not yet sustainable in the long term [29].

We found that, on average, for threatened species in the ISIS network, fewer than 10% of zoos within optimized clusters hold a particular species. Therefore, it would be possible to improve the network within an optimized cluster by increasing the number of zoos that contain individuals of a focal species, managing these collections as a single metapopulation and potentially reducing the distances between zoos. This level of organization could result in zoos focusing on particular CBPs for fewer taxa, rather than having a small number of individuals of many threatened species. This is particularly important since specialization has been shown to increase breeding success [5]. This observation does not mean that zoos should shift their entire collections towards one or a few at-risk species, since responsible zoos have other conservation goals such being centers for education and research [21]. Rather, it means that zoos within a particular region can most efficiently increase their conservation contribution by developing collectively managed CBPs devoted towards a smaller number of focal species.

The proportion of threatened species that exceed a threshold metapopulation size of 250 individuals is rather low, ranging from only 9% for birds to 18% for mammals. However, the percentages of species reaching the threshold of >50 individuals range from 27% for amphibians to ∼45% for the other three classes. ISIS zoos have only a small number of threatened species for which population sizes are above 1,000 individuals. Although this number has been suggested as an appropriate threshold over which genetic diversity should be maintained [30], most of the species that have been recovered from CBPs come from populations below 30 to 20 individuals (see [9]). In this sense, it is important not to underestimate the potential of some of these ISIS populations. Nonetheless, the zoo community should aim at providing populations to CBPs that can ensure genetic and demographic sustainability [31]. Additionally, CBPs should not be implemented only when species have reached dramatically low numbers, at which point their chances of success are lower [3]. Our results stress that, for many species, appropriate management and coordination within an optimized cluster of zoos can potentially increase their numbers to at least 250 individuals. In addition, it is expected that these population sizes will be larger if we include non-ISIS member institutions. However, it is important to stress that the successful management of CBPs as metapopulations requires the collaboration and coordination of zoos within a global network such as ISIS.

### Management and policy recommendations

1. The zoo community should identify potential zoo clusters for the conservation of prioritized species. Particularly for small-bodied species, zoos could potentially hold a large number of individuals within a particular region. Although the clusters should ideally be in close proximity to the species' natural habitat, most ISIS zoos are currently located away from major biodiversity hotspots [3] and therefore, zoos' support of breeding centers within the native range may be an option.
2. Cluster-level integrated management plans should be implemented to ensure the coordination of CBPs with habitat conservation and other *in situ* efforts (One Plan approach [15], [16]. For this, the development of greater coordination among zoos using networks such as ISIS, together with conservation NGOs, academic and governmental institutions, will be essential (see [4]).
3. Species-specific clusters should ideally be replicated to minimize the potential impact of catastrophic events.
4. Cross-border management policies for zoos should be modified. In this sense, the management of CBPs by clusters can facilitate treaties for the management of particular focal species. To ensure sustainable metapopulations it will require more than the coordination and will of the zoo and conservation community. Although we acknowledge the need for public health management and vigilance against illegal trade, the development of cross-border management policies will be key to achieving successful CBPs. For example, CITES could award special permits to facilitate movement of targeted individuals within CBP clusters.

### Conclusions

Global change represents an unprecedented challenge for the maintenance of biodiversity [32]–[34]. It is expected that even under the most optimistic impact and adaptation scenarios, a great number of species may require the integration of a suite of conservation actions, including CBPs. Furthermore, species that have no likelihood of *in situ* persistence for the foreseeable future represent an additional conservation challenge. For example, under current global warming trends, most polar and some montane species are likely to fall into this category [35], in addition to species whose habitat will be lost by urbanization [36]. As a result, deciding which species could be part of successful CBPs and which institutions should modify their collections to become part of a particular cluster needs to be the result of a sound prioritization approach. Furthermore, simply holding these species, even in numbers above the minimum 50 individuals, is not sufficient [31], and CBPs need to be integrated with other aspects of species conservation, such as habitat protection and restoration, eradication of invasive species and population management [3]–[5], [15], [18]. Our results show that, in the zoo network, the representation of species that may require CBPs is currently low for most taxa and the spatial distribution of these zoo populations makes management difficult. However, the zoo network already devotes 23% of its collections to threatened species; for mammals, 18% of those reach population sizes above 250 individuals. Zoos in collaboration with other institutions have already saved a number of species from extinction, but it has been mostly opportunistic rather than strategic. If zoos collectively focus on their strength as a global network, they have the potential for the development of integrated conservation programs that include CBPs. To maximize effectiveness, the collaboration of the global zoo network with governmental institutions, regional and international trade authorities, NGOs and academia should be fostered. Such collaborations are already underway, and termed a One Plan approach [16]. However, it is essential to strengthen these institutions' contributions, include special international treaties and collaborations to help slow down current extinction trends.

## Supporting Information

Table S1 Number of individual animals in ISIS zoos in each of the IUCN Red List categories for each taxonomic class of terrestrial vertebrates.
(DOCX)

Table S2 Representation of threatened mammal species for each taxonomic order in ISIS zoos.
(DOCX)

Table S3 Representation of threatened bird species for each taxonomic order in ISIS zoos.
(DOCX)

Table S4 Representation of threatened reptile species for each taxonomic order in ISIS zoos.
(DOCX)

Table S5 Representation of threatened amphibian species for each taxonomic order in ISIS zoos.
(DOCX)
