# Supplementary material for: Zoos through the Lens of the IUCN Red List: A Global Metapopulation Approach to Support Conservation Breeding Programs
Source: PLoS One. 2013 Dec 11;8(12):e80311. doi: 10.1371/journal.pone.0080311 (PMC3859473; doi:10.1371/journal.pone.0080311)
Supplement: Table S1 — Number of individual animals in ISIS zoos in each of the IUCN Red List categories for each taxonomic class of terrestrial vertebrates. (DOCX) [file pone.0080311.s001.docx]

**Table S1.** Number of individual animals in ISIS zoos in each of the IUCN Red List categories (DD = Data Deficient, LC = Least Concern, NT = Near Threatened, VU = Vulnerable, EN = Endangered, CR = Critically Endangered, EW = Extinct in the Wild) for each taxonomic class of terrestrial vertebrates.

| Class | Animals in ISIS zoos according to IUCN Red List categories | | | | | | | Total animals in ISIS zoos | Threatened animals in ISIS zoos |
| --- | --- | --- | --- | --- | --- | --- | --- | --- | --- |
|  | DD | LC | NT | VU* | EN* | CR* | EW |  |  |
| Amphibia | 14 | 10,206 | 2,138 | 1,984 | 1,985 | 7,053 | 5,420 | 28,800 | 11,022 (38.3%) |
| Aves | 194 | 166,916 | 19,430 | 20,863 | 5,104 | 2,625 | 156 | 215,288 | 28,592 (13.3%) |
| Mammalia | 1,429 | 109,555 | 16,643 | 18,660 | 16,580 | 6,791 | 2,065 | 171,723 | 42,031 (24.1%) |
| Reptilia† | 125 | 12,231 | 10,131 | 10,044 | 2,310 | 4,665 | 0 | 39,506 | 17,019 (43.0%) |
| Total | 1,762 | 298,908 | 48,342 | 51,551 | 25,979 | 21,134 | 7,641 | 455,317 | 98,664 (22.0%) |

*VU, EN and CR are collectively referred to as threatened species

†The reptile assessment by IUCN is not yet complete, so these results only refer to the species assessed in the IUCN Red List version 3.2 (2009)
