# Supplementary material for: Zoos through the Lens of the IUCN Red List: A Global Metapopulation Approach to Support Conservation Breeding Programs
Source: PLoS One. 2013 Dec 11;8(12):e80311. doi: 10.1371/journal.pone.0080311 (PMC3859473; doi:10.1371/journal.pone.0080311)
Supplement: Table S4 — Representation of threatened reptile species for each taxonomic order in ISIS zoos. (DOCX) [file pone.0080311.s004.docx]

**Table S4.** Representation of threatened reptile species for each taxonomic order in ISIS zoos: total number of described species per order, number of threatened species, total number of species in ISIS zoos, number of threatened species in ISIS zoos, expected number of species if zoos planned their collections at random, *P*-values and representation of each order in ISIS zoos (low: lower number of species than expected; high: higher number of species than expected; NS: not significant, i.e. number of species as expected) (NT: no threatened species for that order, so the analyses do not apply).

| Order | Total species on Red List | Threatened species on Red List | Total species in ISIS zoos | Threatened species in ISIS zoos | Expected | *P*-value | Representation in ISIS zoos |
| --- | --- | --- | --- | --- | --- | --- | --- |
| Testudines | 212 | 131 | 153 | 104 | 94.702 | 0.001 | High |
| Crocodilya | 23 | 10 | 22 | 10 | 9.570 | 0.570 | NS |
| Rhynchocephalia | 2 | 1 | 2 | 1 | 1.000 | 1.000 | NS |
| Squamata | 1,440 | 327 | 268 | 63 | 60.791 | 0.052 | NS |
